# A data-driven combined prediction method for the demand for intensive care unit healthcare resources in public health emergencies

**DOI:** 10.1186/s12913-024-10955-8

**Published:** 2024-04-17

**Authors:** Weiwei Zhang, Xinchun Li

**Affiliations:** School of Logistics, Beijing Wuzi University, No.321, Fuhe Street, Tongzhou District, Beijing, 101149 China

**Keywords:** Public health emergency, ICU healthcare resource demand, Machine learning, Combined prediction

## Abstract

**Background:**

Public health emergencies are characterized by uncertainty, rapid transmission, a large number of cases, a high rate of critical illness, and a high case fatality rate. The intensive care unit (ICU) is the “last line of defense” for saving lives. And ICU resources play a critical role in the treatment of critical illness and combating public health emergencies.

**Objective:**

This study estimates the demand for ICU healthcare resources based on an accurate prediction of the surge in the number of critically ill patients in the short term. The aim is to provide hospitals with a basis for scientific decision-making, to improve rescue efficiency, and to avoid excessive costs due to overly large resource reserves.

**Methods:**

A demand forecasting method for ICU healthcare resources is proposed based on the number of current confirmed cases. The number of current confirmed cases is estimated using a bilateral long-short-term memory and genetic algorithm support vector regression (BILSTM-GASVR) combined prediction model. Based on this, this paper constructs demand forecasting models for ICU healthcare workers and healthcare material resources to more accurately understand the patterns of changes in the demand for ICU healthcare resources and more precisely meet the treatment needs of critically ill patients.

**Results:**

Data on the number of COVID-19-infected cases in Shanghai between January 20, 2020, and September 24, 2022, is used to perform a numerical example analysis. Compared to individual prediction models (GASVR, LSTM, BILSTM and Informer), the combined prediction model BILSTM-GASVR produced results that are closer to the real values. The demand forecasting results for ICU healthcare resources showed that the first (ICU human resources) and third (medical equipment resources) categories did not require replenishment during the early stages but experienced a lag in replenishment when shortages occurred during the peak period. The second category (drug resources) is consumed rapidly in the early stages and required earlier replenishment, but replenishment is timelier compared to the first and third categories. However, replenishment is needed throughout the course of the epidemic.

**Conclusion:**

The first category of resources (human resources) requires long-term planning and the deployment of emergency expansion measures. The second category of resources (drugs) is suitable for the combination of dynamic physical reserves in healthcare institutions with the production capacity reserves of corporations. The third category of resources (medical equipment) is more dependent on the physical reserves in healthcare institutions, but care must be taken to strike a balance between normalcy and emergencies.

## Introduction

The outbreak of severe acute respiratory syndrome (SARS) in 2003 was the first global public health emergency of the 21st century. From SARS to the coronavirus disease (COVID-19) pandemic at the end of 2019, followed shortly by the monkeypox epidemic of 2022, the global community has witnessed eight major public health events within the span of only 20 years [1]. These events are all characterized by high infection and fatality rates. For example, the number of confirmed COVID-19 cases worldwide is over 700 million, and the number of deaths has exceeded 7 million [2]. Every major public health emergency typically consists of four stages: incubation, outbreak, peak, and decline. During the outbreak and transmission, surges in the number of infected individuals and the number of critically ill patients led to a corresponding increase in the urgent demand for intensive care unit (ICU) medical resources. ICU healthcare resources provide material security for rescue work during major public health events as they allow critically ill patients to be treated, which decreases the case fatality rate and facilitates the prevention and control of epidemics. Nevertheless, in actual cases of prevention and control, the surge in patients has often led to shortages of ICU healthcare resources and a short-term mismatch of supply and demand, which are problems that have occurred several times in different regions. These issues can drastically impact anti-epidemic frontline healthcare workers and the treatment outcomes of infected patients. According to COVID-19 data from recent years, many infected individuals take about two weeks to progress from mild to severe disease. As the peak of severe cases tends to lag behind that of infected cases, predicting the changes in the number of new infections can serve as a valuable reference for healthcare institutions in forecasting the demand for ICU healthcare resources. The accurate forecasting of the demand for ICU healthcare resources can facilitate the rational resource allocation of hospitals under changes in demand patterns, which is crucial for improving the provision of critical care and rescue efficiency. Therefore, in this study, we combined a support vector regression (SVR) prediction model optimized by a genetic algorithm (GA) with bidirectional long-short-term memory (BILSTM), with the aim of enhancing the dynamic and accurate prediction of the number of current confirmed cases. Based on this, we forecasted the demand for ICU healthcare resources, which in turn may enable more efficient resource deployment during severe epidemic outbreaks and improve the precise supply of ICU healthcare resources.

Research on the demand forecasting of emergency materials generally employs quantitative methods, and traditional approaches mainly include linear regression and GM (1,1). Linear regression involves the use of regression equations to make predictions based on data. Sui et al. proposed a method based on multiple regression that aimed to predict the demand for emergency supplies in the power grid system following natural disasters [3]. Historical data was used to obtain the impact coefficient of each factor on emergency resource forecasting, enabling the quick calculation of the demand for each emergency resource during a given type of disaster. However, to ensure prediction accuracy, regression analysis needs to be supported by data from a large sample size. Other researchers have carried out demand forecasting for emergency supplies from the perspective of grey prediction models. Li et al. calculated the development coefficient and grey action of the grey GM (1,1) model using the particle swarm optimization algorithm to minimize the relative errors between the real and predicted values [4]. Although these studies have improved the prediction accuracy of grey models, they mainly involve pre-processing the initial data series without considering the issue of the excessively fast increase in predicted values by traditional grey GM (1,1) models. In emergency situations, the excessively fast increase in predicted values compared to real values will result in the consumption of a large number of unnecessary resources, thereby decreasing efficiency and increasing costs. As traditional demand forecasting models for emergency supplies have relatively poor perfect order rates in demand analysis, which result in low prediction accuracy, they are not mainstream.

At present, dynamic models of infectious diseases and demand forecasting models based on machine learning are at the cutting edge of research. With regard to the dynamic models of infectious diseases, susceptible infected recovered model (SIR) is a classic mathematical model employed by researchers [5–7]. After many years of development, the SIR model has been expanded into various forms within the field of disease transmission, including susceptible exposed infected recovered model (SEIR) and susceptible exposed infected recovered dead model (SEIRD) [8, 9]. Nevertheless, with the outbreak of COVID-19, dynamic models of infectious diseases have once again come under the spotlight, with researchers combining individual and group variables and accounting for different factors to improve the initial models and reflect the state of COVID-19 [10–13]. Based on the first round of epidemic data from Wuhan, Li et al. predicted the time-delay distributions, epidemic doubling time, and basic reproductive number [14]. Upon discovering the presence of asymptomatic COVID-19 infections, researchers began constructing different SEIR models that considered the infectivity of various viral incubation periods, yielding their respective predictions of the inflection point. Based on this, Anggriani et al. further considered the impact of the status of infected individuals and established a transmission model with seven compartments [15]. Efimov et al. set the model parameters for separating the recovered and the dead as uncertain and applied the improved SEIR model to analyze the transmission trend of the pandemic [16]. In addition to analyzing the transmission characteristics of normal COVID-19 infection to predict the status of the epidemic, many researchers have also used infectious disease models to evaluate the effects of various epidemic preventive measures. Lin et al. applied an SEIR model that considered individual behavioral responses, government restrictions on public gatherings, pet-related transmission, and short-term population movements [17]. Cao et al. considered the containment effect of isolation measures on the pandemic and solved the model using Euler’s numerical method [18]. Reiner et al. employed an improved SEIR model to study the impact of non-pharmaceutical interventions implemented by the government (e.g., restricting population movement, enhancing disease testing, and increasing mask use) on disease transmission and evaluated the effectiveness of social distancing and the closure of public spaces [19]. These studies have mainly focused on modeling the COVID-19 pandemic to perform dynamic forecasting and analyze the effectiveness of control measures during the epidemic. Infectious disease dynamics offer good predictions for the early transmission trends of epidemics. However, this approach is unable to accurately estimate the spread of the virus in open-flow environments. Furthermore, it is also impossible to set hypothetical parameters, such as disease transmissibility and the recovery probability constant, that are consistent with the conditions in reality. Hence, with the increase in COVID-19 data, this approach has become inadequate for the accurate long-term analysis of epidemic trends.

Machine learning has shown significant advantages in this regard [20, 21]. Some researchers have adopted the classic case-based reasoning approach in machine learning to make predictions. However, it is not feasible to find historical cases that fully match the current emergency event, so this approach has limited operability. Other researchers have also employed neural network training in machine learning to make predictions. For example, Hamou et al. predicted the number of injuries and deaths, which in turn were used to forecast the demand for emergency supplies [22]. However, this approach requires a large initial dataset and a high number of training epochs, while uncertainty due to large changes in intelligence information can lead to significant errors in data prediction [23–25]. To address these problems, researchers have conducted investigations that account (to varying degrees) for data characterized by time-series and non-linearity and have employed time-series models with good non-linear fitting [26–28]. The use of LSTM to explore relationships within the data can improve the accuracy of predicting COVID-19 to some extent. However, there are two problems with this approach. First, LSTM neural networks require extremely large datasets, and each wave of the epidemic development cycle would be insufficient to support a dataset suitable for LSTM. Second, neural networks involve a large number of parameters and highly complex models and, hence, are susceptible to overfitting, which can prevent them from achieving their true and expected advantages in prediction.

Overall, Our study differs from other papers in the following three ways. First, the research object of this paper focuses on the specific point of ICU healthcare resource demand prediction, aiming to improve the rate of critical care patient treatment. However, past research on public health emergencies has focused more on resource prediction , such as N95 masks, vaccines, and generalized medical supplies during the epidemic , to mitigate the impact of rapid transmission and high morbidity rates. This has led to less attention being paid to the reality of the surge in critically ill patients due to their high rates of severe illness and mortality.

Second, the idea of this paper is to further forecast resource needs based on the projected number of people with confirmed diagnoses, which is more applicable to healthcare organizations than most other papers that only predict the number of people involved. However, in terms of the methodology for projecting the number of people, this paper adopts a combined prediction method that combines regression algorithms and recurrent neural networks to propose a BILSTM-GASVR prediction model for the number of confirmed diagnoses. It capitalizes on both the suitability of SVR for small samples and non-linear prediction as well as the learning and memory abilities of BILSTM in processing time-series data. On the basis of the prediction model for the number of infected cases, by considering the characteristics of ICU healthcare resources, we constructed a demand forecasting model of emergency healthcare supplies. Past public health emergencies are more likely to use infectious disease models or a single prediction model in deep learning. some of the articles, although using a combination of prediction, but also more for the same method domain combination, such as CNN-LSTM, GRU-LSTM, etc., which are all recurrent neural networks.

Third, in terms of specific categorization of resources to be forecasted, considering the specificity of ICU medical resources, we introduce human resource prediction on the basis of previous studies focusing on material security, and classified ICU medical resources into three categories: ICU human resources, drugs and medical equipment. The purpose of this classification is to match the real-life prediction scenarios of public health emergencies and improve the demand forecasting performance for local ICU healthcare resources. Thus, it is easy for healthcare institutions to grasp the overall development of events, optimizing decision-making, and reducing the risk of healthcare systems collapsing during the outbreak stage.

## Methods

In this section, we accomplish the following two tasks. Firstly, we introduce the idea of predicting the number of infected cases and show the principle of the relevant models. Secondly, based on the number of infected cases, ICU healthcare resources are divided into two categories (healthcare workers and healthcare supplies), and their respective demand forecasting models are constructed.

### Prediction model for the number of infected cases

#### GASVR model

Support vector machine (SVM) is a machine-learning language for classification developed by Vapnik [29]. Suppose there are two categories of samples: H1 and H2. If hyperplane H is able to correctly classify the samples into these two categories and maximize the margin between the two categories, it is known as the optimal separating hyperplane (OSH). The sample vectors closest to the OSH in H1 and H2 are known as the support vectors. To apply SVM to prediction, it is essential to perform regression fitting. By introducing the $\varepsilon$-insensitive loss function, SVM can be converted to a support vector regression machine, where the role of the OSH is to minimize the error of all samples from this plane. SVR has a theoretical basis in statistical learning and relatively high learning performance, making it suitable for performing predictions in small-sample, non-linear, and multi-dimensional fields [30, 31].

Assume the training sample set containing $l$ training samples is given by $\{({x}_{i},{y}_{i}),i=\mathrm{1,2},...,l\}$, where ${x}_{i}=[{x}_{i}^{1},{x}_{i}^{2},...,{x}_{i}^{d}{]}^{\rm T}$ and ${y}_{i}\in R$ are the corresponding output values.

Let the regression function be $f(x)=w\Phi (x)+b$, where $\phi (x)$ is the non-linear mapping function. The linear $\varepsilon$-insensitive loss function is defined as shown in formula (1).

1 $$L(f(x),y,\varepsilon )=\left\{\begin{array}{ll}0,\left|y-f(x)\right|\le \varepsilon \\ \left|y-f(x)\right|-\varepsilon ,\left|y-f(x)\right|>\varepsilon \end{array}\right.$$

Among the rest, $f(x)$ is the predicted value returned by the regression function, and $y$ is the corresponding real value. If the error between $f(x)$ and $y$ is ≤ $\varepsilon$, the loss is 0; otherwise, the loss is $\left|y-f(x)\right|-\varepsilon$.

The slack variables ${\xi }_{i}$ and ${\xi }_{i}^{*}$ are introduced, and $w$, $b$ are solved using the following equation as shown in formula (2).

2 $$\begin{array}{c}\mathit{min}\frac{1}{2}{\Vert \omega \Vert }^{2}+C\sum\limits _{i=1}^{l}({\xi }_{i}+{\xi }_{i}^{*})\\ s.t.\left\{\begin{array}{c}{y}_{i}-\omega \phi (x)-b\le \varepsilon +{\xi }_{i}\\ -{y}_{i}+\omega \phi (x)+b\le \varepsilon +{\xi }_{i}^{*}\\ {\xi }_{i}\ge 0,{\xi }_{i}^{*}\ge 0,i=\mathrm{1,2},...l\end{array}\right.\end{array}$$

Among the rest, $C$ is the penalty factor, with larger values indicating a greater penalty for errors > $\varepsilon$; $\varepsilon$ is defined as the error requirement, with smaller values indicating a smaller error of the regression function.

The Lagrange function is introduced to solve the above function and transformed into the dual form to give the formula (3).

3 $$\begin{array}{c}\mathit{max}[-\frac{1}{2}\sum\limits_{i=1}^{l}\sum\limits_{j=1}^{l}({a}_{i}-{a}_{i}^{*})({a}_{j}-{a}_{j}^{*})K({x}_{i},{x}_{j})-\sum\limits_{i=1}^{l}({a}_{i}+{a}_{i}^{*})\varepsilon +\sum\limits_{i=1}^{l}({a}_{i}-{a}_{i}^{*})]\\ s.t.\left\{\begin{array}{c}\sum\limits_{i=1}^{l}({a}_{i}-{a}_{i}^{*})=0\\ 0\le {a}_{i}\le C\\ 0\le {a}_{i}^{*}\le C\end{array}\right.\end{array}$$

Among the rest, $K({x}_{i},{x}_{j})=\Phi ({x}_{i})\Phi ({x}_{j})$ is the kernel function. The kernel function determines the structure of high-dimensional feature space and the complexity of the final solution. The Gaussian kernel is selected for this study with the function $K({x}_{i},{x}_{j})=\mathit{exp}(-\frac{\Vert {x}_{i}-{x}_{j}\Vert }{2{\sigma }^{2}})$.

Let the optimal solution be $a=[{a}_{1},{a}_{2},...,{a}_{l}]$ and ${a}^{*}=[{a}_{1}^{*},{a}_{2}^{*},...,{a}_{l}]$ to give the formula (4) and formula (5).

4 $$f(x)={\omega }^{*}\Phi (x)+{b}^{*}=\sum\limits_{i=1}^{l}({a}_{i}-{a}_{i}^{*})\Phi ({x}_{i})\Phi (x)+{b}^{*}=\sum\limits_{i=1}^{l}({a}_{i}-{a}_{i}^{*})K({x}_{i},{x}_{j})+{b}$$

5 $$b^\ast\;=\;\frac1{N_{nsv}}\;\left\{\,\sum\limits_{i=1}^l\left[y_i\;-\;\sum\limits_{x_i\in sv}\;\left(a_i\;-\;a_i^\ast\right)\;K\left(x_i,x_j\right)-\varepsilon\right]\;+\;\sum\limits_{0<a_i<c}^l\left[y_i-\sum\limits_{x_i\in sv}\;\left(a_i-a_i^\ast\right)\;K\left(x_i,x_j\right)+\varepsilon\right]\right\}$$

Among the rest, ${N}_{nsv}$ is the number of support vectors.

In sum, the regression function is as shown in formula (6).

6 $$f(x)={\omega }^{*}\Phi (x)+{b}^{*}=\sum\limits_{i=1}^{l}({a}_{i}-{a}_{i}^{*})\Phi ({x}_{i})\Phi (x)+{b}^{*}=\sum\limits_{i=1}^{l}({a}_{i}-{a}_{i}^{*})K({x}_{i},{x}_{j})+{b}$$

when some of the parameters are not 0, the corresponding samples are the support vectors in the problem. This is the principle of SVR. The values of the three unknown parameters (penalty factor C, ε -insensitive loss function, and kernel function coefficient $\sigma )$, can directly impact the model effect. The penalty factor C affects the degree of function fitting through the selection of outliers in the sample by the function. Thus, excessively large values lead to better fit but poorer generalization, and vice versa. The ε value in the ε-insensitive loss function determines the accuracy of the model by affecting the width of support vector selection. Thus, excessively large values lead to lower accuracy that does not meet the requirements and excessively small values are overly complex and increase the difficulty. The kernel function coefficient $\sigma$ determines the distribution and range of the training sample by controlling the size of inner product scaling in high-dimensional space, which can affect overfitting.

Therefore, we introduce other algorithms for optimization of the three parameters in SVR. Currently the commonly used algorithms are 32and some heuristic algorithms. Although the grid search method is able to find the highest classification accuracy, which is the global optimal solution. However, sometimes it can be time-consuming to find the optimal parameters for larger scales. If a heuristic algorithm is used, we could find the global optimal solution without having to trace over all the parameter points in the grid. And GA is one of the most commonly used heuristic algorithms, compared to other heuristic algorithms, it has the advantages of strong global search, generalizability, and broader blending with other algorithms.

**Fig. 1:**
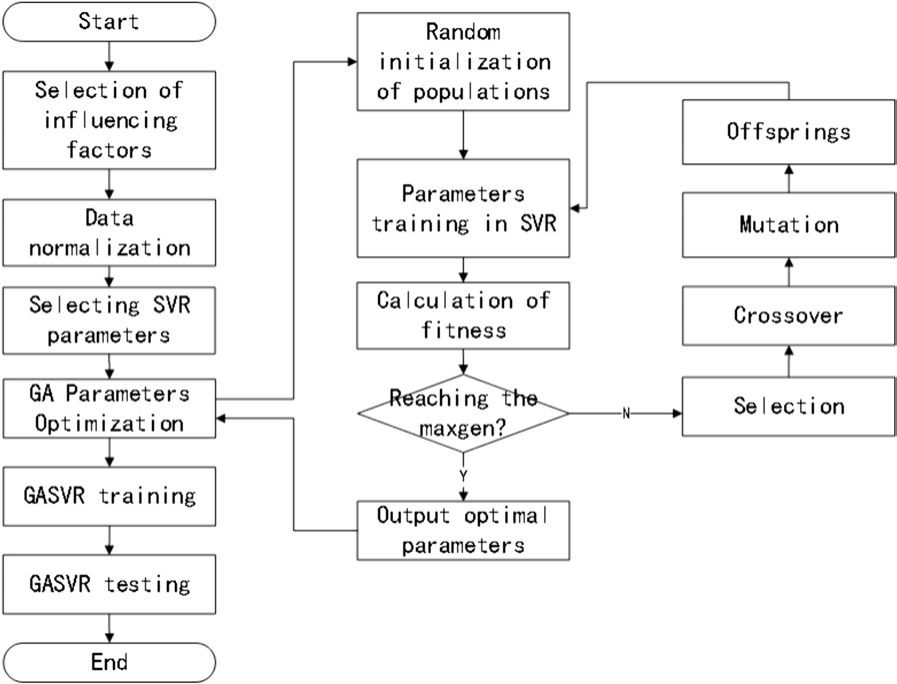
Prediction process of the GASVR model

Given these factors, we employ a GA to encode and optimize the relevant parameters of the model. The inputs are the experimental training dataset, the Gaussian kernel function expression, the maximum number of generations taken by the GA, the accuracy range of the optimized parameters, the GA population size, the fitness function, the probability of crossover, and the probability of mutation. The outputs are the optimal penalty factor C, ε-insensitive loss function parameter $\varepsilon ,$ and optimal Gaussian kernel parameter $\sigma$ of SVR, thus achieving the optimization of SVR. The basic steps involved in GA optimization are described in detail below, and the model prediction process is shown in Fig. 1.

1. Population initialization

The three parameters are encoded using binary arrays composed of 0–1 bit-strings. Each parameter consisted of six bits, and the initial population is randomly generated. The population size is set at 60, and the number of iterations is 200.

(2) Fitness calculation

In the same dataset, the K-fold cross-validation technique is used to test each individual in the population, with K = 5. K-fold cross validation effectively avoids the occurrence of model over-learning and under-learning. For the judgment of the individual, this paper evaluates it in terms of fitness calculations. Therefore, combining the two enables the effective optimization of the model’s selected parameters and improves the accuracy of regression prediction.

Fitness is calculated using the mean error method, with smaller mean errors indicating better fitness. The fitness function is shown in formula (7) [32].

7 $$f=\frac{1}{n}\sum\limits_{i=1}^{n}\left|\frac{{Y}_{i}-\stackrel{\wedge }{{Y}_{i}}}{{Y}_{i}}\right|)$$

The individual’s genotype is decoded and mapped to the corresponding parameter value, which is substituted into the SVR model for training. The parameter optimization range is 0.01 ≤ C ≤ 100, 0.1 ≤$\sigma$ ≤ 20, and 0.001 ≤ ε ≤ 1.

(3) Selection: The selection operator is performed using the roulette wheel method.
(4) Crossover: The multi-point crossover operator, in which two chromosomes are selected and multiple crossover points are randomly chosen for swapping, is employed. The crossover probability is set at 0.9.
(5) Mutation: The inversion mutation operator, in which two points are randomly selected and the gene values between them are reinserted to the original position in reverse order, is employed. The mutation probability is set at 0.09.
(6) Decoding: The bit strings are converted to parameter sets.

The parameter settings of the GASVR model built in this paper are shown in Table 1.

**Table 1:** The GASVR model related parameters

Parameters	Values
$NP$	60
$\mathit{max}g{\text{en}}$	200
${P}_{c}$	0.9
${P}_{m}$	0.09
$$C$$	[0.01,100]
$$\sigma$$	[0.1,20]
$$\varepsilon$$	[0.001,1]

#### BILSTM model

**Fig. 2:**
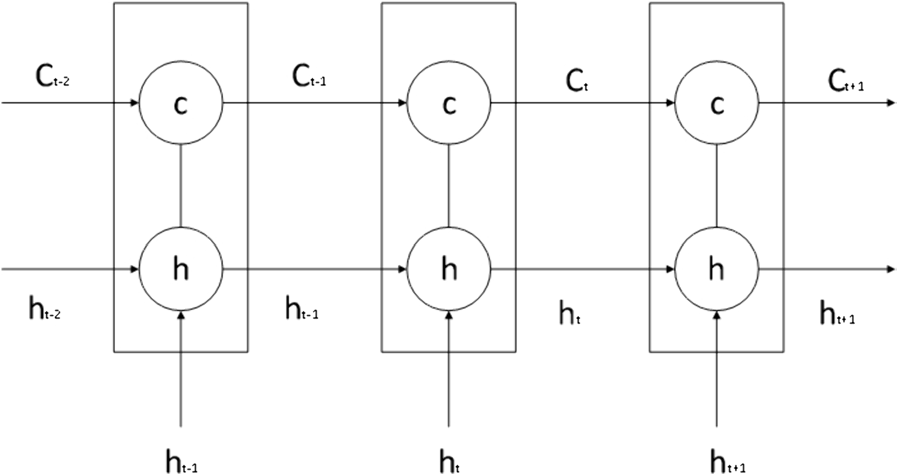
Schematic diagram of the LSTM model

The LSTM model is a special recurrent neural network algorithm that can remember the long-term dependencies of data series and has an excellent capacity for self-learning and non-linear fitting. LSTM automatically connects hidden layers across time points, such that the output of one time point can arbitrarily enter the output terminal or the hidden layer of the next time point. Therefore, it is suitable for the sample prediction of time-series data and can predict future data based on stored data. Details of the model are shown in Fig. 2.

LSTM consists of a forget gate, an input gate, and an output gate.

The forget gate combines the previous and current time steps to give the output of the sigmoid activation function. Its role is to screen the information from the previous state and identify useful information that truly impacts the subsequent time step. The equation for the forget gate is shown in formula (8).

8 $${f}_{t}=\sigma ({b}_{f}+{W}_{f}[{h}_{t-1},{x}_{t}])$$

Among the number, $W_{f}$ is the weight of the forget gate, ${b}_{f}$ is the bias, $\sigma$ is the sigmoid activation function, ${f}_{t}$ is the output of the sigmoid activation function, $t-1$ is the previous time step, $t$ is the current time step, and ${x}_{t}$ is the input time-series data at time step $t$.

The input gate is composed of the output of the sigmoid and tanh activation functions, and its role is to control the ratio of input information entering the information of a given time step. The equation for the input gate is shown in formula (9).

9 $${i}_{t}=\sigma ({b}_{i}+{W}_{i}[{h}_{t-1},{x}_{t}])$$

Among the number, ${W}_{i}$ is the output weight of the input gate, ${i}_{t}$ is the output of the sigmoid activation function, ${b}_{i}$ and ${b}_{C}$ are the biases of the input gate, and ${W}_{C}$ is the output of the tanh activation function.

The role of the output gate is to control the amount of information output at the current state, and its equation is shown in formula (10).

10 $${o}_{t}=\sigma ({b}_{o}+{W}_{o}[{h}_{t-1},{x}_{t}])$$

Among the number, ${W}_{o}$ is the weight of ${o}_{t}$, and ${b}_{o}$ is the bias of the output gate.

The values of the above activation functions $\sigma$ and tanh are generally shown in formulas (11) and (12).

11 $$\sigma (x)=\frac{1}{1+{e}^{-x}}$$

12 $$\mathit{tan}h(x)=\frac{{e}^{x}-{e}^{-x}}{{e}^{x}+{e}^{-x}}$$

${C}_{t}$ is the data state of the current time step, and its value is determined by the input information of the current state and the information of the previous state. It is shown in formula (13).

13 $${C}_{t}=\widetilde{{C}_{t}}\times {i}_{t}+{C}_{t-1}\times {f}_{t}$$

Among the number, $\widetilde{{C}_{t}}=\mathit{tan}h({W}_{c}[{h}_{t-1},{x}_{t}]+{b}_{c})$.

${h}_{t}$ is the state information of the hidden layer at the current time step, ${h}_{t}={o}_{t}\times \mathit{tan}h({c}_{t})$.Each time step ${T}_{n}$ has a corresponding state ${C}_{t}$. By undergoing the training process, the model can learn how to modify state ${C}_{t}$ through the forget, output, and input gates. Therefore, this state is consistently passed on, implying that important distant information will neither be forgotten nor significantly affected by unimportant information.

**Fig. 3:**
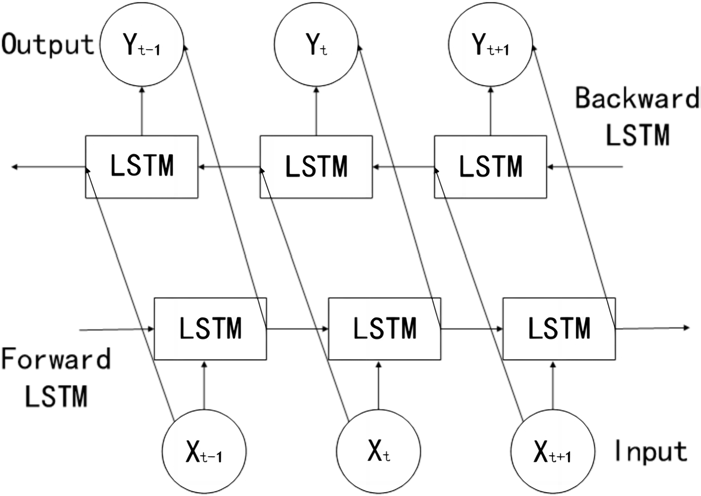
Schematic diagram of the BILSTM model

The above describes the principle of LSTM, which involves forward processing when applied. BILSTM consists of two LSTM networks, one of which processes the input sequence in the forward direction (i.e., the original order), while the other inputs the time series in the backward direction into the LSTM model. After processing both LSTM networks, the outputs are combined, which eventually gives the output results of the BILSTM model. Details of the model are presented in Fig. 3.

Compared to LSTM, BILSTM can achieve bidirectional information extraction of the time-series and connect the two LSTM layers onto the same output layer. Therefore, in theory, its predictive performance should be superior to that of LSTM. In BILSTM, the equations of the forward hidden layer($\overrightarrow{{h}_{t}}$) , backward hidden layer($\overleftarrow{{h}_{t}}$) , and output layer(${o}_{t}$) are shown in formulas (14) , (15) and (16).

14 $$\overrightarrow{{h}_{t}}=\sigma (\overrightarrow{{W}_{xh}}{x}_{t}+\overrightarrow{{W}_{hh}}\overrightarrow{{h}_{t-1}}+\overrightarrow{{b}_{h}})$$

15 $$\overleftarrow{{h}_{t}}=\sigma (\overleftarrow{{W}_{xh}}{x}_{t}+\overleftarrow{{W}_{hh}}\overleftarrow{{h}_{t-1}}+\overleftarrow{{b}_{h}})$$

16 $${o}_{t}=\overrightarrow{{W}_{xh}}\overrightarrow{h}+\overleftarrow{{W}_{hy}}\overleftarrow{h}+{b}_{y}$$

The parameter settings of the BILSTM model built in this paper are shown in Table 2.

**Table 2:** The BILSTM model related parameters

Parameters	Values
epoch	30
batch size	57
layers	2
hidden size	128
time step	10
dropout	0.1
learning rate	0.006
optimizer	Adam

#### Informer model

The Informer model follows the compiler-interpreter architecture in the Transformer model, and based on this, structural optimizations have been made to reduce the computational time complexity of the algorithm and to optimize the output form of the interpreter. The two optimization methods are described in detail next.

With large amounts of input data, neural network models can have difficulty capturing long-term interdependencies in sequences, which can produce gradient explosions or gradient vanishing and affect the model's prediction accuracy. Informer model solves the existential gradient problem by using a ProbSparse Self-attention mechanism to make more efficient than conventional self-attention.

The value of Transformer self-attention is shown in formula (17).

17 $${\text{Attention}}(\text{Q, K, V})= \text{SoftMax} (\frac{Q{K}^{\rm T}}{\sqrt{d}})V$$

Among them, $Q\in {R}^{{L}_{Q}\times d}$ is the query matrix, $K\in {R}^{{L}_{K}\times d}$ is the key matrix, and $V\in {R}^{{L}_{V}\times d}$ is the value matrix, which are obtained by multiplying the input matrix X with the corresponding weight matrices ${W}^{Q}$,${W}^{K}$,${W}^{V}$ respectively, and d is the dimensionality of Q, K, and V. Let ${q}_{i}$,${k}_{i}$,$v_{i}$ represent the ith row in the Q, K, V matrices respectively, then the ith attention coefficient is shown in formula (18) as follows.

18 $${\text{Attention}}({q}_{i}\text{, K, V})=\sum\limits_{j}\frac{k({q}_{i},{K}_{j})}{{\sum }_{l}k({q}_{i},{K}_{l})}{v}_{i}={E}_{p({k}_{j}|{q}_{i})}[{v}_{j}]$$

Therein, $p({k}_{j}|{q}_{i})$ denotes the traditional Transformer's probability distribution formula, and $k({q}_{i},{K}_{l})$ denotes the asymmetric exponential sum function. Firstly, q=1 is assumed, which implies that the value of each moment is equally important; secondly, the difference between the observed distribution and the assumed one is evaluated by the KL scatter, if the value of KL is bigger, the bigger the difference with the assumed distribution, which represents the more important this moment is. Then through inequality $ln{L}_{k}\le M({q}_{i},K)\le {\mathit{max}}_{j}\left\{\frac{{q}_{i}{k}_{j}^{\rm T}}{\sqrt{d}}\right\}-\frac{1}{{L}_{k}}{\sum }_{j=1}^{{L}_{k}}\left\{\frac{{q}_{i}{k}_{j}^{\rm T}}{\sqrt{d}}\right\}+ln{L}_{k}$,$M({q}_{i},K)$ is transformed into $\overline{M}({q}_{i},K)$. According to the above steps, the ith sparsity evaluation formula is obtained as shown in formula (19) [33].

19 $$\left\{\begin{array}{c}M({q}_{i},K)=\mathit{ln}\sum\limits_{j=1}^{{L}_{k}}{e}^{\frac{{q}_{i}{k}_{j}^{\rm T}}{\sqrt{d}}}-\frac{1}{{L}_{k}}\sum\limits_{j=1}^{{L}_{k}}\frac{{q}_{i}{k}_{j}^{\rm T}}{\sqrt{d}}\\ \overline{M}({q}_{i},K)={\mathit{max}}_{j}\left\{\frac{{q}_{i}{k}_{j}^{\rm T}}{\sqrt{d}}\right\}-\frac{1}{{L}_{k}}\sum\limits_{j=1}^{{L}_{k}}\left\{\frac{{q}_{i}{k}_{j}^{\rm T}}{\sqrt{d}}\right\}\end{array}\right.$$

One of them, $M({q}_{i},K)$ denotes the ith sparsity measure; $\overline{M}({q}_{i},K)$ denotes the ith approximate sparsity measure; ${L}_{k}$ is the length of query vector. $TOP-u$ quantities of $\overline{M}$ are selected to form $\overline{Q}$ , $\overline{Q}$ is the first u sparse matrices, and the final sparse self-attention is shown in Formula (20). At this point, the time complexity is still $O({n}^{2})$, and to solve this problem, only l moments of M2 are computed to reduce the time complexity to $O(L\cdot \mathit{ln}(L))$.

20 $${\text{Attention}}(\text{Q, K, V})= \text{SoftMax} (\frac{\overline{Q}{K}^{\rm T}}{\sqrt{d}})V$$

**Fig. 4:**
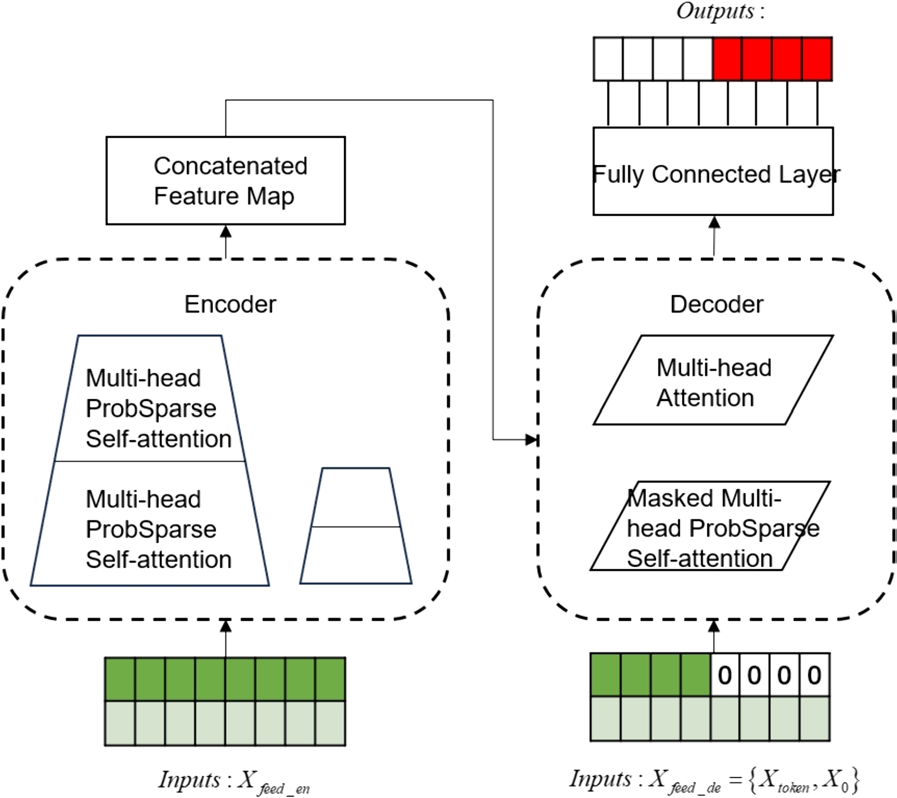
Informer uses a generative decoder to obtain long sequence outputs

Informer uses a generative decoder to obtain long sequence outputs.Informer uses the standard decoder architecture shown in Fig. 4, in long time prediction, the input given to the decoder is shown in formula (21).

21 $${X}_{de}^{t}=Concat({X}_{token}^{t},{X}_{0}^{t})\in {R}^{({L}_{token}+{L}_{y})\times {d}_{\mathit{mod}el}}$$

Therein, ${X}_{de}^{t}$ denotes the input to the decoder; ${X}_{token}^{t}\in {R}^{({L}_{token}+{L}_{y})\times {d}_{\mathit{mod}el}}$ is the dimension of the encoder output, which is the starting token without using all the output dimensions; ${X}_{0}^{t}\in {R}^{({L}_{token}+{L}_{y})\times {d}_{\mathit{mod}el}}$ is the dimension of the target sequence, which is uniformly set to 0; and finally the splicing input is performed to the encoder for prediction.

The parameter settings of Informer model created in this paper are shown in Table 3.

**Table 3:** The Informer model related parameters

Parameters	Values
d_model	512
n_heads	16
s_layers	2
batch_size	57
d_ff	2048
train_epochs	6
dropout	0.1
learning rate	0.0001
patience	6
optimizer	Adam
embed	Time

#### BILSTM-GASVR combined prediction model

SVR has demonstrated good performance in solving problems like finite samples and non-linearity. Compared to deep learning methods, it offers faster predictions and smaller empirical risks. BILSTM has the capacity for long-term memory, can effectively identify data periodicity and trends, and is suitable for the processing of time-series data. Hence, it can be used to identify the effect of time-series on the number of confirmed cases. Given the advantages of these two methods in different scenarios, we combined them to perform predictions using GASVR, followed by error repair using BILSTM. The basic steps for prediction based on the BILSTM-GASVR model are as follows:

1. Normalization is performed on the initial data.
2. The GASVR model is applied to perform training and parameter optimization of the data to obtain the predicted value $\widehat{{y}_{i}}$.
3. After outputting the predicted value of GASVR, the residual sequence between the predicted value and real data is extracted to obtain the error ${\gamma }_{i}$ (i.e., ${\gamma }_{i}={y}_{i}-\widehat{{y}_{i}}$).
4. The BILSTM model is applied to perform training of the error to improve prediction accuracy. The BILSTM model in this paper is a multiple input single output model. Its inputs are the true and predicted error values ${\gamma }_{i}$ and its output is the new error value $\widehat{{\gamma }_{i}}$ predicted by BILSTM.
5. The final predicted value is the sum of the GASVR predicted value and the BILSTM residual predicted value (i.e., ${Y}_{i}=\widehat{{y}_{i}}+\widehat{{\gamma }_{i}}$).

The parameter settings of the BILSTM-GASVR model built in this paper are shown in Table 4.

**Table 4:** The BILSTM-GASVR model related parameters

Parameters	Values
$NP$	60
$\mathit{max}g{\text{en}}$	200
${P}_{c}$	0.9
${P}_{m}$	0.09
$$C$$	[0.01,100]
$$\sigma$$	[0.1,20]
$$\varepsilon$$	[0.001,1]
epoch	30
batch size	57
layers	1
hidden size	64
time step	6
dropout	0.1
learning rate	0.001
optimizer	Adam

#### Model testing criteria

To test the effect of the model, the prediction results of the BILSTM-GASVR model are compared to those of GASVR, LSTM, BILSTM and Informer. The prediction error is mainly quantified using three indicators: mean squared error (MSE), root mean squared error (RMSE), and correlation coefficient ($R^{2}$). Their respective equations are shown in formulas (22), (23) and (24).

22 $$MES=\frac{1}{n}\sum\limits_{i=1}^{n}({Y}_{i}-\widehat{{Y}_{i}}{)}^{2}$$

23 $$RMES=\sqrt{\frac{1}{n}\sum\limits_{i=1}^{n}({Y}_{i}-\widehat{{Y}_{i}}{)}^{2}}$$

24 $${R}^{2}=\frac{({\sum }_{i=1}^{N}({Y}_{i}-\widehat{{Y}_{i}})(\widehat{{Y}_{i}}-\overline{\widehat{{Y}_{i}}}){)}^{2}}{{\sum }_{i=1}^{N}({Y}_{i}-\widehat{{Y}_{i}}{)}^{2}{\sum }_{i=1}^{N}(\widehat{{Y}_{i}}-\overline{\widehat{{Y}_{i}}}{)}^{2}}$$

### Demand forecasting model of ICU healthcare resources

ICU healthcare resources can be divided into human and material resources. Human resources refer specifically to the professional healthcare workers in the ICU. Material resources, which are combined with the actual consumption of medical supplies, can be divided into consumables and non-consumables. Consumables refer to the commonly used drugs in the ICU, which include drugs for treating cardiac insufficiency, vasodilators, anti-shock vasoactive drugs, analgesics, sedatives, muscle relaxants, anti-asthmatic drugs, and anticholinergics. Given that public health emergencies have a relatively high probability of affecting the respiratory system, we compiled a list of commonly used drugs for respiratory diseases in the ICU (Table 5).

**Table 5:** Partial list of common ICU drugs for respiratory diseases

Effect	Drug type	Name of typical drugs
Antibiotics	β-lactams	Penicillins, cephalosporins
	Quinolones	Levofloxacin, moxifloxacin
	Macrolides	Erythromycin, clarithromycin
	Aminoglycosides	Streptomycin, gentamicin
Anti-asthmatic drugs	β_2_ adrenergic receptor agonists	Albuterol, formoterol
	Theophyllines	Aminophylline, diprophylline
	Hormones	Beclomethasone dipropionate, prednisolone, dexamethasone
	Anticholinergics	Atropine, tiotropium bromide
Antitussives and expectorants	Mucolytics	Ambroxol hydrochloride, erdosteine
	Nausea-stimulating expectorants	Guaifenesin
	Other expectorants	Myrtol
	Central antitussives	Dextromethorphan, pentoxyverine, cloperastine
	Peripheral antitussives	Benzonatate, noscapine, lidocaine
Antipyretics	Compound antitussives	Aspirin
	Salicylates	Acetaminophen
	Acetanilides	Ibuprofen

Non-consumables refer to therapeutic medical equipment, including electrocardiogram machines, blood gas analyzers, electrolyte analyzers, bedside diagnostic ultrasound machines, central infusion workstations, non-invasive ventilators, invasive ventilators, airway clearance devices, defibrillators, monitoring devices, cardiopulmonary resuscitation devices, and bedside hemofiltration devices.

The demand forecasting model of ICU healthcare resources constructed in this study, as well as its relevant parameters and definitions, are described below. ${R}_{ij}^{n}$ is the forecasted demand for the $i$ ^th^ category of resources on the $n$ ^th^ day in region $j$. ${Y}_{j}^{n}$ is the predicted number of current confirmed cases on the $n$ ^th^ day in region $j$. ${M}_{j}^{n}$ is the number of ICU healthcare workers on the $n$ ^th^ day in region $j$, which is given by the following formula: number of healthcare workers the previous day + number of new recruits − reduction in number the previous day, where the reduction in number refers to the number of healthcare workers who are unable to work due to infection or overwork. In general, the number of ICU healthcare workers should not exceed 5% of the number of current confirmed cases (i.e., it takes the value range [0, $Y_{j}^{n}$×5%]). $U_{i}$ is the maximum working hours or duration of action of the $i$ ^th^ resource category within one day. ${A}_{j}$ is the number of resources in the $i$ ^th^ category allocated to patients (i.e., how many units of resources in the $i$ ^th^ category is needed for a patient who need the $i$ ^th^ unit of the given resource). ${\varphi }_{i}$ is the demand conversion coefficient (i.e., the proportion of the current number of confirmed cases who need to use the $i$ ^th^ resource category). ${C}_{ij}^{n}$ is the available quantity of material resources of the $i$ ^th^ category on the $n$ ^th^ day in region $j$. At the start, this quantity is the initial reserve, and once the initial reserve is exhausted, it is the surplus from the previous day. The formula for this parameter is given as follows: available quantity from the previous day + replenishment on the previous day − quantity consumed on the previous day, where if ${C}_{ij}^{n}$ is a negative number, it indicates the amount of shortage for the given category of resources on the previous day.

In summary, the demand forecast for emergency medical supplies constructed in this study is shown in formula (25).

25 $${R}_{ij}^{n}=\left\{\begin{array}{c}\frac{({Y}_{j}^{n}{\varphi }_{i}-\frac{{M}_{j}^{n}}{{A}_{j}})}{{A}_{j}}\times \frac{24}{{U}_{i}},i=1\\ {Y}_{j}^{n}{\varphi }_{i}\frac{24}{{U}_{i}}-{C}_{ij}^{n},i=2\\ \frac{({Y}_{j}^{n}{\varphi }_{i}-\frac{{C}_{ij}^{n}}{{A}_{j}})}{{A}_{j}},i=3\end{array}\right.$$

The number of confirmed cases based on data-driven prediction is introduced into the demand forecasting model for ICU resources to forecast the demand for the various categories of resources. In addition to the number of current confirmed cases, the main variables of the first demand forecasting model for human resources are the available quantity and maximum working hours. The main variable of the second demand forecasting model for consumable resources is the number of units consumed by the available quantity. The main variable of the third model for non-consumable resources is the allocated quantity. These three resource types can be predicted using the demand forecasting model constructed in this study.

## Results

### Prediction of the number of current infected cases

The COVID-19 situation in Shanghai is selected for our experiment. A total of 978 entries of epidemic-related data in Shanghai between January 20, 2020, and September 24, 2022, are collected from the epidemic reporting platform. This dataset is distributed over a large range and belongs to a right-skewed leptokurtic distribution. The specific statistical description of data is shown in Table 6. Part of the data is shown in Table 7.

**Table 6:** Statistical description of the results of the indicators

Indicator	Value
mean	693.8
median	76
mode	59 and 67
standard deviation	3022.9
kurtosis	41.1
skewness	6.3
minimum	1
maximum	24609

**Table 7:** Status of COVID-19 cases in Shanghai between January 20, 2020, and December 6, 2022

Date	Current number of confirmed cases	Number of newly confirmed cases	Number of local newly confirmed cases	Number of newly imported cases (positive)	Number of asymptomatic cases	Number of newly recovered cases	Number of new deaths	Cumulative number of confirmed cases	Cumulative number of recovered cases	Cumulative number of deaths
2020/1/20	1	1	0	28	0	0	0	1	0	0
2020/1/21	9	8	0	22	0	0	0	9	0	0
2020/1/22	16	7	0	13	0	0	0	16	0	0
2020/1/23	20	4	0	22	0	0	0	20	0	0
2020/1/24	32	13	0	10	0	1	0	33	1	0
2020/1/25	38	7	0	10	0	0	1	40	1	1
2020/1/26	51	13	0	20	0	0	0	53	1	1
2020/1/27	62	13	0	7	0	2	0	66	3	1
2020/1/28	75	14	0	10	0	1	0	80	4	1
......	......	......	......	......	......	......	......	......	......	......
2022/9/16	101	9	0	9	1	11	0	63987	63291	595
2022/9/17	104	16	0	16	0	13	0	64003	63304	595
2022/9/18	107	11	0	11	3	8	0	64014	63312	595
2022/9/19	102	5	0	5	1	10	0	64019	63322	595
2022/9/20	106	8	0	8	0	4	0	64027	63326	595
2022/9/21	110	9	0	9	0	5	0	64036	63331	595
2022/9/22	106	15	0	15	1	19	0	64051	63350	595
2022/9/23	106	15	0	15	0	15	0	64066	63365	595
2022/9/24	102	9	0	9	0	13	0	64075	63378	595

And we divided the data training set and test set in an approximate 8:2 ratio, namely, 798 days for training (January 20, 2020 to March 27, 2022) and 180 days for prediction (March 28, 2022 to September 24, 2022).

Due to the large difference in order of magnitude between the various input features, directly implementing training and model construction would lead to suboptimal model performance. Such effects are usually eliminated through normalization. In terms of interval selection, [0, 1] reflects the probability distribution of the sample, whereas [-1, 1] mostly reflects the state distribution or coordinate distribution of the sample. Therefore, [-1, 1] is selected for the normalization interval in this study, and the processing method is shown in formula (26).

26 $${X}_{new}=2\times \frac{X-{X}_{min}}{{Xmin}_{max}-1}$$

Among the rest, $X$ is the input sample, ${X}_{min}$ and ${X}_{max}$ are the minimum and maximum values of the input sample, and ${X}_{new}$ is the input feature after normalization.

In addition, we divide the data normalization into two parts, considering that the amount of data in the training set is much more than the test set in the real operating environment. In the first step, we normalize the training set data directly according to the above formula; in the second step, we normalize the test data set using the maximum and minimum values of the training data set.

**Fig. 5:**
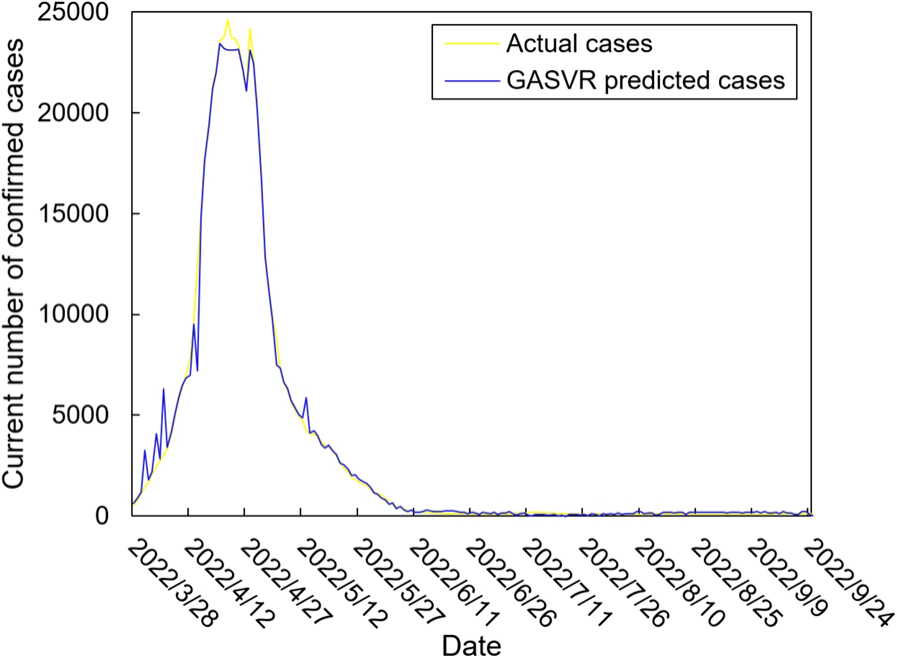
The prediction result of the GASVR model

**Fig. 6:**
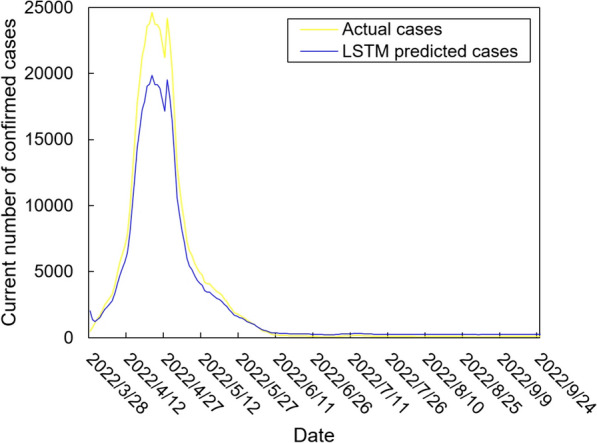
The prediction result of the LSTM model

**Fig. 7:**
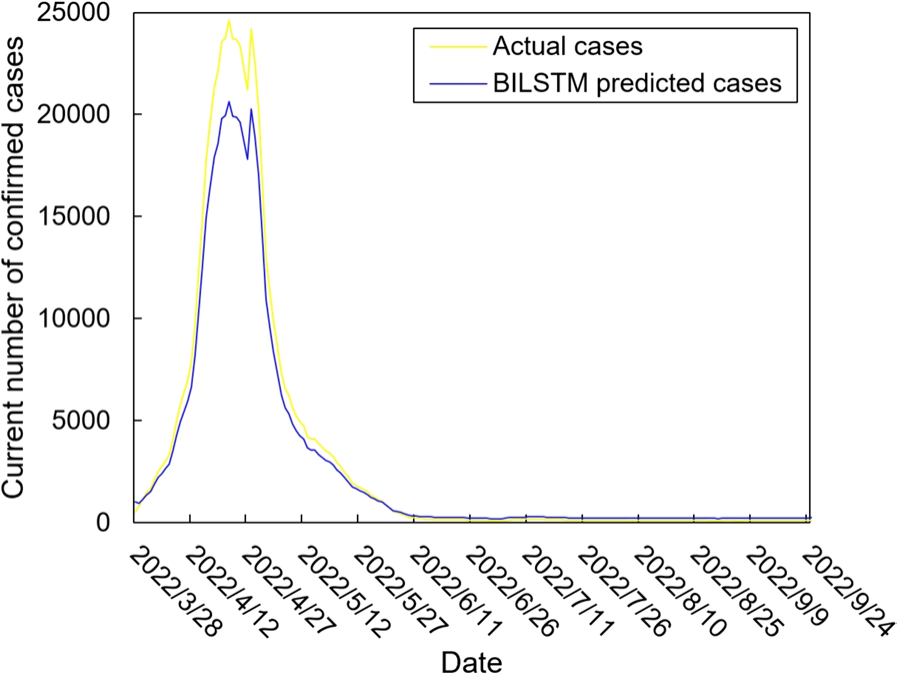
The prediction result of the BILSTM model

**Fig. 8:**
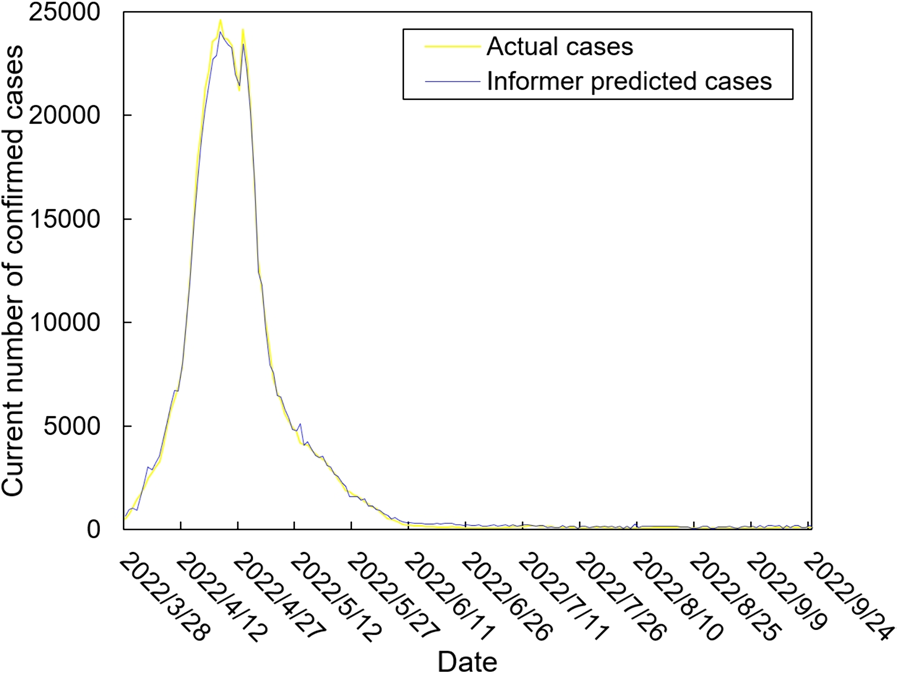
The prediction result of the Informer model

**Fig. 9:**
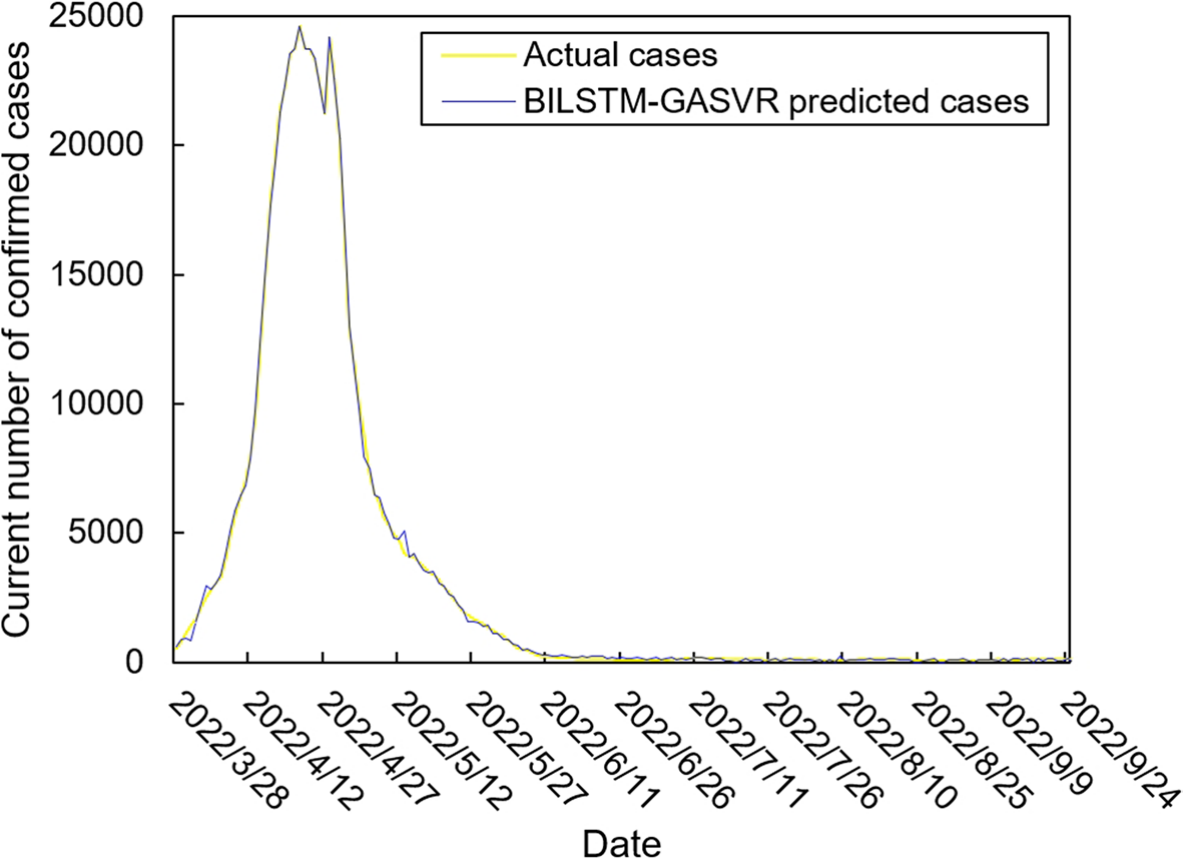
The prediction result of the BILSTM-GASVR model

The values of the preprocessed data are inserted into the GASVR, LSTM, Informer, BILSTM models and the BILSTM-GASVR model is constructed. Figures 5, 6, 7, 8 and 9 show the prediction results. From Figs. 5, 6, and 7, it can be seen that in terms of data accuracy, GASVR more closely matches the real number of infected people relative to BILSTM and LSTM. Especially in the most serious period of the epidemic in Shanghai (April 17, 2022 to April 30, 2022), the advantage of the accuracy of the predicted data of GASVR is even more obvious, which is due to the characteristics of GASVR for small samples and nonlinear prediction. However, in the overall trend of the epidemic, BILSTM and LSTM, which have the ability to learn and memorize to process time series data, are superior. It is clearly seen that in April 1, 2022-April 7, 2022 and May 10, 2022-May 15, 2022, there is a sudden and substantial increase in GASVR in these two time phases, and a sudden and substantial decrease in April 10, 2022-April 14, 2022. These errors also emphasize the stability of BILSTM and LSTM, which are more closely matched to the real epidemic development situation in the whole process of prediction, and the difference between BILSTM and LSTM prediction is that the former predicts data more accurately than the latter, which is focused on the early stage of prediction as well as the peak period of the epidemic. Informer is currently an advanced time series forecasting method. From Fig. 8, it can be seen that the prediction data accuracy and the overall trend of the epidemic are better than the single prediction models of GASVR, LSTM and BILSTM. However, Informer is more suitable for long time series and more complex and large prediction problems, so the total sample size of less than one thousand cases is not in the comfort zone of Informer model. Figure 9 shows that the BILSTM-GASVR model constructed in this paper is more suitable for this smaller scale prediction problem, with the best prediction results, closest to the actual parameter (number of current confirmed cases), demonstrating small sample and time series advantages. In Short, the prediction effect of models is ranked as follows: BILSTM-GASVR> Informer> GASVR> BILSTM> LSTM.

The values of the three indicators (MSE, RMSE, and correlation coefficient ${R}^{2}$) for the five models are shown in Table 8. MSE squares the error so that the larger the model error, the larger the value, which help capture the model's prediction error more sensitively. RMSE is MSE with a root sign added to it, which allows for a more intuitive representation of the order of magnitude difference from the true value. ${R}^{2}$ is a statistical indicator used to assess the overall goodness of fit of the model, which reflects the overall consistency of the predicted trend and does not specifically reflect the degree of data. The results in the Table 8 are consistent with the prediction results in the figure above, while the ranking of MSE, RMSE, and ${R}^{2}$ are also the same (i.e., BILSTM-GASVR> Informer> GASVR> BILSTM> LSTM).

**Table 8:** Comparison of the predictive performance of different models

Model	MSE	RMSE	$R^{2}$
BILSTM-GASVR	17238	131.294	0.9996
Informer	44022	209.821	0.9994
BILSTM	569878	754.903	0.9858
LSTM	1837130	1355.408	0.9544
GASVR	289712	538.249	0.9928

In addition, we analyze the five model prediction data using significance tests as a way of demonstrating whether the model used is truly superior to the other baseline models. The test dataset with kurtosis higher than 4 does not belong to the approximate normal distribution, so parametric tests are not used in this paper. Given that the datasets predicted by each of the five models are continuous and independent datasets, this paper uses the Kruskal-Wallis test, which is a nonparametric test. The test steps are as follows.

1. Determine hypotheses (H0, H1) and significance level ($\alpha$).
2. For each data set, all its sample data are combined and ranked from smallest to largest. Then find the number of data items (${n}_{i}$), rank sum (${R}_{i}$) and mean rank of each group of data respectively.
3. Based on the rank sum, the test statistic (H) is calculated for each data set in the Kruskal-Wallis test. The specific calculation is shown in formula (27).

27 $$H=\frac{12}{N(N+1)}\sum\limits_{i=1}^{m}\frac{{R}_{i}^{2}}{{n}_{i}}-3(N+1)$$

(4) According to the test statistic and degrees of freedom, find the corresponding p-value in the Kruskal-Wallis distribution table. Based on the P-value, determine whether the original hypothesis is valid.

In the significance test, we set the significance setting original hypothesis (H0) as there is no significant difference between the five data sets obtained from the five predictive models. We set the alternative hypothesis (H1) as there is a significant difference between the five data sets obtained from the five predictive models. At the same time, we choose the most commonly used significance level taken in the significance test, namely 0.05. In this paper, multiple comparisons and two-by-two comparisons of the five data sets obtained from the five predictive models are performed through the SPSS software. The results of the test show that in the multiple comparison session, P=0.001<0.05, so H0 is rejected, which means that the difference between the five groups of data is significant. In the two-by-two comparison session, BILSTM-GASVR is less than 0.05 from the other four prediction models. The specific order of differences is Informer < GASVR < BILSTM < LSTM, which means that the BILSTM-GASVR prediction model does get a statistically significant difference between the dataset and the other models.

In summary, combined prediction using the BILSTM-GASVR model is superior to the other four single models in various aspects in the case study analysis of Shanghai epidemic with a sample size of 978.

### Demand forecasting of ICU healthcare resources

Combined with the predicted number of current infected cases, representatives are selected from the three categories of resources for forecasting. The demand for nurses is selected as the representative for the first category of resources.

In view of the fact that there are currently no specific medications that are especially effective for this public health emergency, many ICU treatment measures involved helping patients survive as their own immune systems eliminated the virus. This involved, for example, administering antibiotics when patients developed a secondary bacterial infection. glucocorticoids are used to temporarily suppress the immune system when their immune system attacked and damaged lung tissues causing patients to have difficulty breathing. extracorporeal membrane oxygenation (ECMO) is used for performing cardiopulmonary resuscitation when patients are suffering from cardiac arrest. In this study, we take dexamethasone injection (5 mg), a typical glucocorticoid drug, as the second category of ICU resources (i.e., drugs); and invasive ventilators as the third category of ICU resources (i.e., medical equipment).

During the actual epidemic in Shanghai, the municipal government organized nine critical care teams, which are stationed in eight municipally designated hospitals and are dedicated to the treatment of critically ill patients. In this study, the ICU nurses, dexamethasone injections, and invasive ventilators in Shanghai are selected as the prediction targets and introduced into their respective demand forecasting models. Forecasting of ICU healthcare resources is then performed for the period from March 28, 2022, to April 28, 2022, as an example. Part of the parameter settings for the three types of resources are shown in Tables 9, 10, and 11, respectively.

**Table 9:** Parameter setting of ICU nurses

Parameter	Value
${U}_{i}$	12
${\varphi }_{i}$	0.13
${M}_{j}^{n}$	1000
${A}_{j}$	0.5

**Table 10:** Parameter setting of dexamethasone injections

Parameter	Value
${U}_{i}$	36
${\varphi }_{i}$	0.22
${C}_{ij}^{n}$	30000

**Table 11:** Parameter setting of invasive ventilators

Parameter	Value
${A}_{j}$	1
$\varphi$	0.11
${C}_{ij}^{n}$	1300

Table 12 shows the forecasting results of the demand for ICU nurses, dexamethasone injections, and invasive ventilators during the epidemic wave in Shanghai between March 28, 2022, and April 28, 2022.

**Table 12:** Demand forecasting of the three categories of ICU healthcare resources

Date	Demand for ICU nurses (person)	Demand for dexamethasone injections (Piece)	Demand for invasive ventilators (Piece)
2022/3/28	0	0	0
2022/3/29	0	0	0
2022/3/30	0	0	0
2022/3/31	0	1870	0
2022/4/1	0	2320	0
2022/4/2	0	2490	0
2022/4/3	110	3050	0
2022/4/4	200	3340	0
2022/4/5	490	4440	0
2022/4/6	820	4800	0
2022/4/7	1090	5490	0
2022/4/8	1310	5010	110
2022/4/9	1530	4960	230
2022/4/10	970	4750	230
2022/4/11	1140	4520	250
2022/4/12	1480	5520	310
2022/4/13	2250	6090	390
2022/4/14	2320	6840	580
2022/4/15	2490	7930	600
2022/4/16	3360	10120	750
2022/4/17	3120	11330	1000
2022/4/18	2270	13550	1110
2022/4/19	2150	14980	580
2022/4/20	2060	14870	770
2022/4/21	2430	14300	240
2022/4/22	2520	14000	240
2022/4/23	2490	13360	0
2022/4/24	1740	12040	0
2022/4/25	830	10500	0
2022/4/26	0	10400	0
2022/4/27	0	11030	0
2022/4/28	0	11020	0

For the first category (i.e., ICU nurses), human resource support is only needed near the peak period, but the supply could not be replenished immediately. In the early stages, Shanghai could only rely on the nurses’ perseverance, alleviating the shortage of human resources by reducing the number of shifts and increasing working hours. This situation persisted until about April 10 and is only resolved when nurses from other provinces and regions successively arrived in Shanghai.

The second category of ICU resources is drugs, which are rapidly consumed. The pre-event reserve of 30,000 dexamethasone injections could only be maintained for a short period and is fully consumed during the outbreak. Furthermore, daily replenishment is still needed, even when the epidemic has passed its peak and begun its decline.

The third category is invasive ventilators, which are non-consumables. Thus, the reserve lasted for a relatively long period of time in the early stages and did not require replenishment after its maximum usage during the peak period.

## Discussion

Demand forecasting models are constructed based on the classification of healthcare resources according to their respective features. We choose ICU nurses, dexamethasone injections, and invasive ventilators as examples, and then forecast demand for the epidemic wave in Shanghai between March 28, 2022, and April 28, 2022. The main conclusions are as follows:

1. A long period of time is needed to train ICU healthcare workers who can independently be on duty, taking at least one year from graduation to entering the hospital, in addition to their requiring continuous learning, regular theoretical training, and the accumulation of clinical experience during this process. Therefore, for the first category of ICU healthcare resources, in the long term, healthcare institutions should place a greater emphasis on their talent reserves. Using China as an example, according to the third ICU census, the ratio of the number of ICU physicians to the number of beds is 0.62:1 and the ratio of the number of nurses to the number of beds is 1.96:1, which are far lower than those stipulated by China itself and those of developed countries. Therefore, a fundamental solution is to undertake proactive and systematic planning and construction to ensure the more effective deployment of human resources in the event of a severe outbreak. In the short term, healthcare institutions should focus on the emergency expansion capacity of their human resources. In case there are healthcare worker shortages during emergencies, the situation can be alleviated by summoning retired workers back to work and asking senior medical students from various universities to help in the hospitals to prevent the passive scenario of severely compressing the rest time of existing staff or waiting for external aid. However, it is worth noting that to ensure the effectiveness of such a strategy of using retired healthcare workers or senior students of university medical faculties, it is necessary for healthcare organizations to provide them with regular training in the norm, such as organizing 2-3 drills a year, to ensure the professionalism and proficiency of healthcare workers who are temporarily and suddenly put on the job. At the same time, it is also necessary to fully mobilize the will of individuals. Medical institutions can provide certain subsidies to retired health-care workers and award them with honorable titles. For senior university medical students, volunteer certificates are issued and priority is given to their internships, so that health-care workers can be motivated to self-realization through spiritual and material rewards.
2. Regarding the second category of ICU resources (i.e., drugs), healthcare institutions perform the subdivision of drug types and carry out dynamic physical preparations based on 15–20% of the service recipient population for clinically essential drugs. This will enable a combination of good preparedness during normal times and emergency situations. In addition, in-depth collaboration with corporations is needed to fully capitalize on their production capacity reserves. This helps medical institutions to be able to scientifically and rationally optimize the structure and quantity of their drug stockpiles to prevent themselves from being over-stressed. Yet the lower demand for medicines at the end of the epidemic led to the problem of excess inventory of enterprises at a certain point in time must be taken into account. So, the medical institutions should sign a strategic agreement on stockpiling with enterprises, take the initiative to bear the guaranteed acquisition measures, and consider the production costs of the cooperative enterprises. These measures are used to truly safeguard the enthusiasm of the cooperative enterprises to invest in the production capacity.
3. Regarding the third category of ICU resources (i.e., medical equipment), large-scale medical equipment cannot be rapidly mass-produced due to limitations in the capacity for emergency production and conversion of materials. In addition, the bulk procurement of high-end medical equipment is also relatively difficult in the short term. Therefore, it is more feasible for healthcare institutions to have physical reserves of medical equipment, such as invasive ventilators. However, the investment costs of medical equipment are relatively high. Ventilators, for example, cost up to USD $50,000, and subsequent maintenance costs are also relatively high. After all, according to the depreciable life of specialized hospital equipment, the ventilator, as a surgical emergency equipment, is depreciated over five years. And its depreciation rate is calculated at 20% annually for the first five years, which means a monthly depreciation of $835. Thus, the excessively low utilization rate of such equipment will also impact the hospital. Healthcare institutions should, therefore, conduct further investigations on the number of beds and the reserves of ancillary large-scale medical equipment to find a balance between capital investment and patient needs.

The limitations of this paper are reflected in the following three points. Firstly, in the prediction of the number of infections, the specific research object in this paper is COVID-19, and other public health events such as SARS, H1N1, and Ebola are not comparatively analyzed. The main reason for this is the issue of data accessibility, and it is easier for us to analyze events that have occurred in recent years. In addition, using the Shanghai epidemic as a specific case may be more representative of the epidemic situation in an international metropolis with high population density and mobility. Hence, it has certain regional limitations, and subsequent studies should expand the scope of the case study to reflect the characteristics of epidemic transmission in different types of urban areas and enhance the generalizability.

Secondly, the main emphasis of this study is on forecasting the demand for ICU healthcare resources across the entire region of the epidemic, with a greater focus on patient demand during public emergencies. Our aims are to help all local healthcare institutions more accurately identify changes in ICU healthcare resource demand during this local epidemic wave, gain a more accurate understanding of the treatment demands of critically ill patients, and carry out comprehensive, scientifically based decision-making. Therefore, future studies can examine individual healthcare institutions instead and incorporate the actual conditions of individual units to construct multi-objective models. In this way, medical institutions can further grasp the relationship between different resource inputs and the recovery rate of critically ill patients, and achieve the balance between economic and social benefits.

Finally, for the BILSTM-GASVR prediction method, in addition to the number of confirmed diagnoses predicted for an outbreak in a given region, other potential applications beyond this type of medium-sized dataset still require further experimentation. For example, whether the method is suitable for procurement planning of a certain supply in production management, forecasting of goods sales volume in marketing management, and other long-period, large-scale and other situations.

## Conclusion

Within the context of major public health events, the fluctuations and uncertainties in the demand for ICU resources can lead to large errors between the healthcare supply and actual demand. Therefore, this study focuses on the question of forecasting the demand for ICU healthcare resources. Based on the number of current confirmed cases, we construct the BILSTM-GASVR model for predicting the number of patients. By comparing the three indicators (MSE, MAPE, and correlation coefficient $R^{2}$) and the results of the BILSTM, LSTM, and GASVR models, we demonstrate that our model have a higher accuracy. Our findings can improve the timeliness and accuracy of predicting ICU healthcare resources and enhance the dynamics of demand forecasting. Hence, this study may serve as a reference for the scientific deployment of ICU resources in healthcare institutions during major public events.

Given the difficulty in data acquisition, only the Shanghai epidemic dataset is selected in this paper, which is one of the limitations mentioned in Part 4. Although the current experimental cases of papers in the same field do not fully conform to this paper, the results of the study cannot be directly compared. However, after studying the relevant reviews and the results of the latest papers, we realize that there is consistency in the prediction ideas and prediction methods [34, 35]. Therefore, we summarize the similarities and differences between the results of the study and other research papers in epidemic forecasting as shown below.

Similarities: on the one hand, we all characterize trends in the spread of the epidemic and predict the number of infections over 14 days. On the other hand, we all select the current mainstream predictive models as the basis and combine or improve them. Moreover, we all use the same evaluation method (comparison of metrics such as MSE and realistic values) to evaluate the improvements against other popular predictive models.

Differences: on the one hand, other papers focus more on predictions at the point of the number of patients, such as hospitalization rate, number of infections, etc. This paper extends the prediction from the number of patients to the specific healthcare resources. This paper extends the prediction from the number of patients to specific healthcare resources. We have divided the medical resources and summarized the demand regularities of the three types of information in the epidemic, which provides the basis for decision-making on epidemic prevention to the government or medical institutions. On the other hand, in addition to the two assessment methods mentioned in the same point, this paper assesses the performance of the prediction methods with the help of significance tests, which is a statistical approach to data. This can make the practicality of the forecasting methodology more convincing.

## Data Availability

The datasets used and/or analysed during the current study available from the corresponding author on reasonable request.
